# General practice interventions to reduce cardiovascular disease risk in patients with severe mental illness: A scoping review

**DOI:** 10.1371/journal.pone.0349303

**Published:** 2026-05-13

**Authors:** Aswath Krishna Muthuraman, Nandakumar Ravichandran, Niamh Murphy, John Broughan, Eleni Niarchou, Brian O’Donoghue, Joseph Gallagher, Kenneth McDonald, Janis Morrissey, Walter Cullen

**Affiliations:** 1 School of Medicine, University College Dublin, Dublin, Ireland; 2 Clinical Research Centre, School of Medicine, University College Dublin, Dublin, Ireland; 3 St Vincent’s University Hospital, Dublin, Ireland; 4 Irish Heart Foundation, Dublin, Ireland; University of Missouri School of Medicine, UNITED STATES OF AMERICA

## Abstract

**Background:**

People with severe mental illness (SMI), including bipolar disorder, major depression, and schizophrenia, face significantly increased morbidity and mortality from cardiovascular disease (CVD). Common CVD risk factors in these populations comprise health behaviours such as smoking and poor diet, and physical factors, including diabetes mellitus, obesity and dyslipidaemia. Thus, there is a need to identify interventions to prevent CVD in SMI patients, for which general practice may be ideal for delivery. This scoping review aimed to explore the interventions to reduce CVD risk in patients with SMI in primary care, with a focus on general practice.

**Methods:**

This scoping review was guided by Arksey and O’Malley’s six-step methodological framework, barring step six – consultation. A systematic search was performed across four electronic databases: PubMed, Embase, APA PsycINFO and CINAHL, following the Preferred Reporting Items for Systematic Reviews and Meta-analyses extension for Scoping Reviews (PRISMA-ScR) guidelines. Narrative synthesis was conducted to identify key themes informed by the Popay et al. framework.

**Results:**

A total of 10 studies were included in the final analysis. Five themes were identified, namely (1) Primary Care Interventions, (2) Intervention Implementation, (3) Collaborative or Intermediary Structures, (4) Barriers and Facilitators, and (5) Participant Experiences and Viewpoints. Identified intervention types included tailored behavioural change interventions, the patient centred medical home model, clinical tool use and provider education. Promising aspects of interventions included effective staff training, collaborative structures and peer support involvement. Challenges to implementation included patient mental health symptoms impacting ability to attend sessions, lack of knowledge and experience among staff of working with SMI patients, and resource constraints, including time and workload concerns.

**Conclusion:**

This scoping review highlights a research gap regarding primary care interventions to reduce CVD risk in SMI patients, as only 10 relevant studies were identified published from 2015 to July 2025 in the English language. However, aspects of existing literature, such as promising intervention features, implementation barriers and feedback for consideration were also identified. Future research regarding this topic could address identified barriers and feedback points. Further randomised controlled trials assessing clinical-effectiveness and cost-efficiency of interventions in primary care settings may also be required.

## Introduction

### Mental illness and cardiovascular disease

Severe mental illness (SMI) is a term referring to “an enduring mental illness that is often so debilitating that the person’s ability to engage in functional and occupational activities is severely impaired” [1]. SMI encompasses several disorders, however this review will focus on bipolar disorder, schizophrenia and major depression [1–3]. Regarding point prevalence, a 2022 study estimated about 11.5% of the Irish population have major depression, and another study from 1990 estimated prevalence of schizophrenia of 0.39% across three Irish counties [4,5]. SMI populations have an average lifespan 10–17.5 years shorter than the general population, with approximately two-thirds of deaths attributable to somatic factors, and cardiovascular disease (CVD) being a major addressable cause of mortality [2,6]. Young adults with SMI are also estimated to have a significantly higher 30-year CVD risk compared with those who do not live with SMI [7]. Furthermore, CVD-related mortality is approximately twice as high in SMI populations and contributes to the widening mortality gap between SMI and general populations over time [8]. A variety of factors may be responsible for this association, including higher rates of physical and behavioural cardiometabolic risk factors such as obesity, diabetes mellitus, alcohol abuse and smoking in SMI populations, as well as antipsychotic related metabolic effects [9,10]. Furthermore, primary care cardiometabolic risk screening rates in SMI populations appear to be suboptimal in SMI populations, relative to both the general population and clinical guideline recommendations [11,12]. Financial incentivisation may improve screening rates, but not necessarily cardiometabolic risk management, highlighting management as a discrete issue [13].

### Role of primary care in CVD risk management

Regarding management of this excess CVD risk, primary care may be an ideal environment [14–16]. Primary care refers to the first point of contact within the healthcare system, encompassing a broad range of community-based services delivered by multidisciplinary professionals. Its structure, funding, and the professionals involved may vary across countries. In many settings, including Ireland and the United Kingdom (UK), general practice constitutes a central component of primary care and is typically delivered by general practitioners (GPs). Terminology varied across studies, with some using ‘primary care’ and others ‘general practice’, reflecting differences in healthcare systems. For consistency, the terms ‘primary care’ and ‘general practice’ are used interchangeably in this review, reflecting variation in terminology across included studies. Current NICE guidelines recommend primary care physical health check-ups for SMI patients at least annually, with risk management following general population guidelines, including statin use for blood lipid control or cardioprotective diet modification [15]. In the UK, financial incentivisation for physical health checks in primary care was introduced for people with SMI in 2004 through the Quality and Outcomes Framework (QOF), ensuring that a care plan is in place and provide annual screening for physical health for this population [17]. Similarly, in Ireland, the Health Service Executive (HSE) introduced financial incentivisation for primary care cardiometabolic monitoring of patients with SMI history as part of the Opportunistic Case Finding programme in the Chronic Disease Management strategy [18]. However, a report from August 2024 found that only 3.9% of patients assessed in the programme until then had a history of SMI, suggesting potential barriers to reaching or engaging this population [18]. Furthermore, a HSE public mental health briefing from 2015 estimated that mental health problems had an overall economic cost of €3 billion in Ireland, indicating a need for more cost-effective interventions addressing mental health problems and associated comorbidities, such as CVD risk factors [19]. Thus, the aim of this scoping review was to explore primary care interventions to reduce cardiovascular disease risk in patients with severe mental illness.

## Methods

This scoping review was conducted following Arksey and O’Malley’s six-step methodological framework, barring step six – consultation, due to practical and time limitations [20]. The steps outlined are, (1) Identifying the Research Question, (2) Identifying Relevant Studies, (3) Study Selection, (4) Charting the Data, and (5) Collating, Summarising and Reporting the Results. Narrative synthesis was also performed, informed by the Popay et al framework [21,22].

### Stage 1: Identifying the research question

The purpose of this scoping review was to explore the primary care interventions to reduce cardiovascular disease risk in patients with severe mental illness. Thus, the following research question was constructed:

“What is the primary care or general practice interventions to reduce cardiovascular disease risk in patients with severe mental illness?”

### Stage 2: Identifying relevant studies

A literature search was conducted across the PubMed, Embase, APA PsycINFO and CINAHL databases following the PRISMA-ScR guidelines. The search syntax is presented in Table 1.

**Table 1:** Search syntax.

(“Cardiovascular risk factors”[tiab] OR “Cardiovascular risk”[tiab] OR “Cardiometabolic risk”[tiab] OR “Cardiometabolic disease risk”[tiab] OR “CVD risk”[tiab] OR “CVD risk factors”[tiab] OR “Cardiovascular disease risk factors”[tiab] OR “Cardiovascular disease risk”[tiab] OR smoking[MeSH Terms] OR smok*[tiab] OR LDL[tiab] OR lipids[MeSH Terms] OR lipid*[tiab] OR cholesterol[MeSH Terms] OR diabetes mellitus[MeSH Terms] OR diabet*[tiab] OR alcohol drinking[MeSH Terms] OR alcohol*[tiab] OR hypertension[MeSH Terms] OR hypertensi*[tiab] OR BMI[tiab] OR obesity[MeSH Terms] OR obes*[tiab] OR “body weight”[MeSH Terms]) AND (“severe mental health”[tiab] OR “severe mental illness”[tiab] OR “severe mental disorder”[tiab] OR “severe mental health disorders”[tiab] OR SMI[tiab] OR bipolar disorder[MeSH Terms] OR bipolar[tiab] OR schizophrenia[MeSH Terms] OR schizo*[tiab] OR Major Depressive Disorder [MeSH Terms] OR “major depression”[tiab] OR “major depressive”[tiab]) AND (“Primary Health Care”[MeSH Terms] OR “primary care”[tiab] OR primary healthcare*[tiab] OR general practice[MeSH Terms] OR “General Practice”[MeSH Terms] OR general practice*[tiab] OR family practice*[tiab] OR “GP”[tiab] OR “FM”[tiab] OR “family medicine”[tiab]) AND (screen*[tiab] OR interven*[tiab] OR treat*[tiab] OR manage*[tiab] OR prevent*[tiab] OR therap*[tiab] OR care[tiab])

Studies published between 2015 and July 2025, and in the English language, were included. One source identified via exploratory manual searching of PubMed which met the defined selection criteria was also included in the review. A total of 1785 articles were identified via the initial search strategy and exploratory searching.

### Stage 3: Study Selection

Studies initially identified were screened based on title and abstract, first by AKM, then by NR. Subsequently full-text review was performed, first by AKM, then by NR. Studies deemed to meet the determined inclusion criteria during full-text appraisal were included in the final review and excluded otherwise, upon which both reviewers agreed. Conflicts between AKM and NR were resolved through discussion and arrival at a commonly agreed conclusion.

**Inclusion criteria**

- Adults aged ≥18 years
- Studies involving individuals diagnosed with SMI, defined as schizophrenia spectrum disorders, bipolar disorder, or major depressive disorder with significant functional impairment
- Studies conducted in primary care or general practice settings, or interventions delivered within or coordinated through general practice or primary care
- Studies involving interventions aimed at managing or modifying cardiovascular or cardiometabolic risk factors (e.g., smoking, obesity, diabetes, hypertension, lipid control)
- Interventions including behavioural, service-level, or clinical support components (e.g., care models, provider education, decision-support tools)
- Empirical research studies including quantitative, qualitative and mixed methods studies published between 2015 and 2025 in the English language

**Exclusion criteria**

- Studies involving individuals with established cardiovascular disease
- Studies focusing exclusively on mild or moderate mental illness (e.g., anxiety disorders, mild depression)
- Studies conducted solely in secondary or tertiary care settings, without a primary care or general practice component
- Studies focusing only on cardiovascular risk screening, without a management or intervention component
- Studies focusing solely on treatment of psychiatric symptoms or established cardiovascular disease, without a cardiovascular risk management component
- Pharmacological-only interventions without a behavioural or primary care or general practice delivery component
- Non-empirical studies (e.g., reviews, editorials, thesis) or study protocols without results

**Fig 1:**
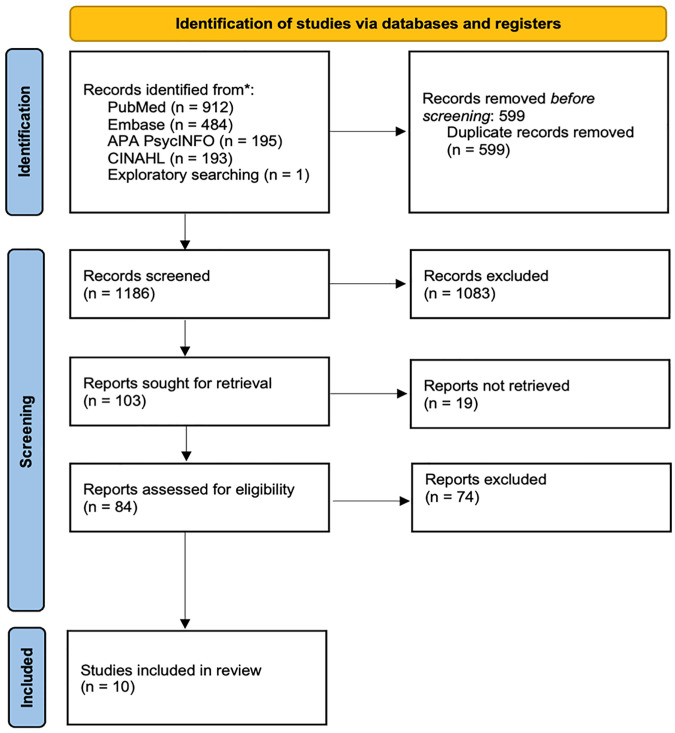
PRISMA-ScR flowchart.

All screening (title/abstract and full-text) was conducted using Covidence adhering to Preferred Reporting Items for Systematic Reviews and Meta-analyses extension for Scoping Reviews (PRISMA-ScR) guidelines [23]. The study selection flowchart is presented as Fig 1.

### Stage 4: Charting the data

Following study selection, relevant data were extracted from the chosen studies by AKR, reviewed by NR and JB. The domains of extraction were the author names, journal of publication, year of publication, study location, study population, study intervention, study aim/ topic, study design and major findings. These data were tabulated to aid presentation and pattern identification for the undertaking of thematic analysis and are included in the results section (see Table 2).

**Table 2:** Characteristics of studies included in final review.

Author (Location)	Journal	Population	Study design & Intervention	Aim/ topic	Major findings
Dronavalli M et al. 2025 (Australia) [24]	The Australian and New Zealand journal of psychiatry	Individuals over 18 years of age with documented mental illness	A Retrospective cohort study on mental health care plans	Investigating if mental health care plans can modify metabolic risk	Mental health care plans were associated with modest but clinically relevant improvements in physical health behaviours, including reductions in daily tobacco smoking and body mass index; however, causal inference is limited due to the observational design.
Evins AE et al. 2023 (United States) [25]	Psychiatric Services	Adults who smoked tobacco and were eligible for Department of Mental Health psychiatric rehabilitation services for SMI	Randomised controlled trial involving Provider education (PE) and community health worker (CHW) support	To determine whether PE and/or CHW support would increase tobacco abstinence compared to care as usual	The combined PE and CHW intervention significantly improved long-term tobacco abstinence over two years compared to PE alone and usual care, indicating the added value of personalised, community-based support.
Gertner AK et al. 2023 (United States) [26]	The Journal of Clinical Psychiatry	Individuals with at least 2 diagnoses out of schizophrenia, schizoaffective disorder or bipolar disorder	Retrospective cohort study on enhanced primary care (EPC) adapted to needs of SMI patients	To assess effect of novel EPC model on cardiometabolic health for people with SMI	EPC was associated with improved cardiometabolic monitoring and better management of risk factors compared to standard care, suggesting that tailored primary care models can address preventive care gaps in SMI populations.
Hassan S et al. 2020 (United Kingdom) [27]	BMC health services research	A sample of participants between 30–75 with SMI, and staff from PRIMROSE trial	Qualitative study on Bespoke nurse-delivered PRIMROSE intervention	To explore barriers and facilitators of implementing PRIMROSE intervention	Implementation was shaped by organisational factors including intervention coherence, staff engagement, and teamwork, highlighting that successful delivery depends on system-level and behavioural factors, rather than intervention design alone.
Lawless, M.E et al. 2016 (United States) [28]	Diabetes Spectrum	Individuals between 25–73 years of age with SMI	Qualitative study on Targeted Training and Illness Management (TTIM) delivered by trained nurse and peer educators	To describe feasibility, acceptability and implementation of nurse-led activities of TTIM intervention	The intervention demonstrated high feasibility and acceptability, with 80% attendance and strong fidelity, although clinical outcomes were not the primary focus, supporting its potential scalability in routine care.
Lee C et al. 2023 (United Kingdom) [29]	BMC Psychiatry	Patients over 18 years of age, with primary SMI diagnosis	Qualitative study on Adjunct Meet your Mentor and Mentor Check In consultations for patients referred to mainstream weight loss programme	To report the development, feasibility and acceptability of the Weight change for people with serious mental illness (WHEEL) intervention	Participants achieved a mean weight loss of 4.1 kg at 12 weeks with high engagement and retention, suggesting mentor-supported interventions can produce short-term behavioural improvements.
Osborn D et al. 2018 (United Kingdom) [30]	Lancet Psychiatry	Patients between 30–75 years of age with SMI, with mean total cholesterol > 5 mmol/L or total:HDL ratio over 4, and one or more additional CVD risk factor	Randomised controlled trial involving Bespoke nurse-delivered PRIMROSE intervention	To evaluate effects of PRIMROSE intervention on decreasing cholesterol concentrations and CVD risk in SMI patients	The intervention showed no significant improvement in cholesterol levels compared to usual care at 12 months; however, it was associated with reduced healthcare costs, indicating economic benefit despite limited clinical effectiveness.
Rossom RC et al. 2023 (United States) [31]	Journal of Clinical Psychiatry	8,922 patients aged 18–75 years with diagnosed SMI and at least one CVD risk factor not at goal	Randomised controlled trial involving Clinical decision support (CDS) tool	To measure impact of CDS tool on total modifiable cardiovascular risk at 12 months	Use of the CDS tool resulted in a modest but statistically significant (4%) reduction in total modifiable cardiovascular risk, supporting the scalability of low-cost digital interventions, although effects on individual risk factors were limited..
Sajatovic, M et al. 2017 (United States) [32]	Psychiatric services: a journal of the American Psychiatric Organisation	200 individuals with SMI and diabetes	Randomised controlled trial involving Targeted Training and Illness Management (TTIM) intervention delivered by trained nurse and peer educators to individuals with SMI	To test whether TTIM intervention in primary care setting will improve SMI symptoms, functional status, general health status and diabetes-specific outcomes	The intervention improved diabetes-related knowledge and aspects of self-management but showed minimal impact on glycaemic control (HbA1c), indicating a gap between behavioural or educational gains and clinical outcomes.
Shaw, P et al. 2024 (United Kingdom) [33]	BMC Health Services Research	Eight patients over 18 years of age with SMI	Qualitative study on PRIMROSE-adapted (PRIMROSE-A) intervention	To determine acceptability of and experiences with PRIMROSE-A via interviews with patients and staff	Participants and staff reported positive perceptions and high acceptability of the intervention; however, the small sample size limits generalisability, and further evaluation is required.

### Stage 5: Collating, summarising and reporting the results

Extracted data were formatted into a table, and further analysis of the chosen articles was performed for narrative synthesis, informed by the Popay et al.et al. framework [21]. This involved four key stages. First, a preliminary synthesis of the included studies was undertaken by organising extracted data into tables and grouping studies based on intervention type and study characteristics. Second, relationships within and between studies were explored by comparing intervention components, implementation strategies, and reported outcomes across studies. Third, thematic analysis was performed to identify recurring patterns and key themes, including intervention types, implementation factors, collaborative structures, and barriers and facilitators. Finally, the robustness of the synthesis was considered through discussion among the team to ensure consistency and validity in the interpretation of findings.

### Stage 6: Consultation

Although a formal consultation process was not undertaken, however, informal stakeholder feedback was sought from general practitioners, a psychiatrist, a chief cardiologist, a director of a non-governmental organisation which supports and campaigns for people who have been affected by heart and strokes throughout their lives and researchers with expertise on mental health and CVD. Their professional feedback and perspectives were used to refine the interpretation of findings and ensure the review’s relevance to clinical practice and CVD risk management in people with SMI. This step aligns with Arksey and O’Malley’s recommendation to enhance the applicability and validity of scoping review findings through stakeholder engagement, even in a limited capacity.

## Results

A total of 1785 articles were initially identified, including one further article identified via exploratory database searching and deemed to meet the eligibility criteria, with 1186 unique articles after removal of 599 duplicates. After title and abstract screening, 1083 articles were deemed irrelevant, resulting in 103 articles moving onto full-text assessment. Following full-text review, 10 articles were included in the final analysis.

### Study characteristics

The 10 identified studies were from Australia (n = 1), the United Kingdom (n = 4) and the United States (n = 5). A range of study designs were adopted, including cohort studies (n = 2), randomised controlled trials (RCTs) (n = 4) and qualitative studies (n = 4).

### Identified themes

The five following key themes were identified via narrative synthesis: 1. Primary Care Interventions, 2. Intervention Implementation, 3. Collaborative or Intermediary Structures, 4. Barriers and Facilitators and 5. Participant Experiences and Viewpoints.

### 1. Primary care interventions

Eight different primary care interventions were described across the 10 studies included in this scoping review. These included six studies on behavioural change interventions [27–30,32,33], two studies on implementation of clinical tools [24,31], one study on provider education intervention [25] and modified primary care model [26]. Intervention targets included general metabolic health [24], cardiometabolic health [26,27,30,31,33] and individual CVD risk factors including diabetes mellitus [28,32], smoking cessation [25] and weight management [29]. The main modes of intervention delivery were in-person only [26,27,30,31], online or telephone calls only [29], mixed with in-person and telehealth [24,28,32,33], and mixed with in-person and written material provision [25].

#### a. Behavioural interventions.

Of the four reported behavioural interventions, the Targeted Training in Illness Management (TTIM) found no significant difference in HbA_1c_ (glycosylated haemoglobin) between the intervention and treatment-as-usual groups after 60 weeks, but did find a significant improvement in diabetes knowledge in the intervention group compared to usual treatment [28,32]. A bespoke cardiovascular risk reduction intervention (PRIMROSE), with emphasis on personalised risk factor management, guiding patients to relevant services, and initiating and continuing indicated pharmacotherapy such as statins, showed no improvements in total blood cholesterol over 12 months compared to usual care [30]. Shaw et al. described an adapted PRIMROSE-A intervention, with further focus on statin prescribing, care integration, and patient mental health, and found it to be positively viewed by patients and staff [33]. Lee et al.et al. described an adjunct mentor support intervention for SMI patients partaking in a mainstream weight loss program, and found it to be feasible and acceptable [29].

#### b. Modified primary care models.

A cohort study by Gertner et al.et al. described an enhanced primary care model, structured as a patient-centred medical home (PCMH), which provided tailored care for SMI patients, with care coordination, peer support and self-management programme provision, where patients were seen, on average, 6 times annually in 30–40 minute appointments [26]. Further modifications included smaller patient panels, as well as specific staff training on treating people with SMI [26]. Primary care providers also had close working relationships with patients’ behavioural care providers in this model, with monthly meetings for discussion of patient needs [26]. This model was associated with decreased HbA_1c_ and systolic blood pressure, as well as increased HbA_1c_, LDL and blood pressure screening rates, compared to standard primary care [26].

#### c. Provider education.

An RCT by Evins et al.et al. evaluated a combined intervention of primary care provider education and community health worker (CHW) support for smoking cessation [25]. The combined intervention improved long-term tobacco abstinence study, highlighting the value of personalised support and primary care provider education [25].

#### d. Clinical tool implementation.

An RCT by Rossom et al.et al. described the implementation of a clinical decision support (CDS) tool that provided individualised CVD risk summaries and treatment recommendations for SMI patients, which was associated with a 4% relative CVD risk reduction compared to control at 12 months, but few significant reductions in individual risk factors such as smoking [31]. A cohort study by Dronavalli et al.et al. found that financially incentivised care plan use was associated with a reduction in daily tobacco smoking rates and excess body weight compared to lack of care plan use [24]. Other studies also incorporated clinical tools, such as care-plans, or information booklets or leaflets for patients, but as components of more complex interventions [28–30].

### 2. Intervention implementation

Aspects pertaining to intervention implementation, such as staff training, patient engagement and staff fidelity, and cost efficiency, were reported across eight studies.

#### a. Staff training.

Staff training was a key component or prerequisite in seven included studies. Evins et al.et al. reported a smoking cessation intervention where education of primary care provider (PCPs) including physicians, nurse prescribers and physician assistants, aimed to increase smoking pharmacotherapy use [25]. PCPs were educated by trained doctoral level staff about the risks and benefits of first-in-line prescriptions, particularly varenicline, for SMI patients through in-person educational outreach and written material provision [25]. The PRIMROSE intervention reported by Osborn et al.et al. and Hassan et al.et al. contained a 2-day training package and provision of a manual for general practice nurses outlining behavioural change interventions informed by the behavioural wheel strategy, regarding risk factor management individualised for patients [27,30]. Nurse practitioner training was also a key component of the PRIMROSE-A intervention described by Shaw et al. [33]. In the TTIM intervention nurses received two days of initial training on a diabetes management intervention for SMI patients from the principal investigators and other team members, including peer educators, as well as communication training from a psychiatrist [28,32]. The provided education was continuous, with quarterly training sessions for discussion of progress and development of skills delivered via in-person meetings, phone calls and email [28]. Peer educator involvement was important for modelling positive SMI and diabetes related behaviours during training in this intervention [28]. In the PCMH described by Gertner et al.et al., staff received specialised training on working with SMI patients, to better understand aspects of care unique to SMI patients, and to learn how to de-escalate crisis situations [26].

#### b. Engagement and fidelity.

Patient attendance was mentioned in three studies and was found to be generally acceptable. Osborn et al.et al. reported that 46% of patients attended 6 or more appointments out of 12, Lee et al. that 12 out 16 patients attended over half of the weekly check-ins, and Lawless et al. that 61% of patients attended all sessions, for each respective intervention [28–30]. Staff fidelity to intervention delivery was mentioned in two studies. Osborn et al. reported moderate adherence, with 67.7% of PRIMROSE intervention components delivered to protocol, though statin initiation was still low in both the treatment and usual care group, and Lawless et al.et al. reported near complete fidelity to the manualised TTIM intervention content, apart from some sessions lasting longer than the allocated 90 minutes [28,30].

#### c. Cost efficiency.

Cost efficiency was reported in only one study, with Osborn et al.et al. reporting that PRIMROSE was on average £895 cheaper per patient than usual treatment over 12 months, primarily due to reduced psychiatric admission costs [30]. Rossom et al. reported that CDS tool implementation incurred a much smaller cost per patient than more intensive interventions, potentially due to its automated nature, requiring minimal maintenance when guidelines are updated, but no figures were provided [31].

### 3. Collaborative or intermediary structures

Collaborative or intermediary structures alongside primary care physicians was an integral component of five interventions and was mentioned in seven studies. Key workers included nurses, healthcare assistants, peer educators and psychologists.

#### a. Nurse practitioners and healthcare assistants.

Nurse practitioners were integral to intervention delivery in five included studies, with health care assistants also delivering interventions in two of the aforementioned studies. The TTIM intervention involved trained nurse educators who delivered group-based sessions, supported personalised care plan formation, educated patients about diabetes self-management, and communicated with patients’ care providers [28,32]. The PRIMROSE and PRIMROSE-A interventions respectively were primarily provided by nurse practitioners, as well as health care assistants, who delivered behavioural change interventions during appointments with SMI patients [30,33]. They aided in aspects such as personalised care plan formation, goal setting, treatment adherence monitoring, guiding patients to relevant supportive services, and initiating pharmacological therapies were indicated [30,33]. A registered nurse was also part of the multidisciplinary team of the PCMH reported by Gertner et al., but their roles were not elucidated further [26].

#### b. Peer support.

The provision of peer support for patients, by people with lived SMI experience, was reported in four included studies. The TTIM intervention involved peer educators who shared personal experiences about diabetes management and SMI with patients, worked closely with nurse educators and modelled effective self-management interventions [28,32]. In the PRIMROSE-A intervention, coaching sessions were offered by a peer-coach with lived experience of SMI, involving discussion of personal experiences and goals [33]. Two trained peer specialists were also involved in the multidisciplinary team of the enhanced primary care model described by Gertner et al., though their roles were not elucidated [26].

#### c. Mentor support.

The WHEEL intervention described by Lawless et al. involved knowledgeable mentors of different professions, such as a graduate psychologist, who offered weekly scheduled calls with SMI patients enrolled in a mainstream weight management programme [29]. Mentors provided adjunct practical support to patients, and helped locate and activate memberships for the mainstream programme [29].

### 4. Barriers and facilitators

Barriers and facilitators to intervention implementation were mentioned in four studies, at the level of the patient, staff and logistics (time, resources, etc.).

#### a. Patient-level barriers and facilitators.

Lee et al. identified patient fear of judgement and unfamiliar or social situations due to hallucinations negatively impacting their desire for social interaction as a barrier to patient attendance, with paranoia also identified as a barrier [29]. Fatigue related to antipsychotics was also reported to negatively impact patient ability to attend sessions [29]. Hassan et al. reported that health care plan use was viewed as both a facilitator and barrier by patients, with some reporting problems with repetitiveness or challenges filing them in, and others finding them helpful to track progress [27]. A strong therapeutic relationship between patient and carer was identified as key facilitator for patient engagement across three studies, by increasing patient willingness to engage [27,29,33]. The availability of a telehealth option was identified as a facilitator of patient engagement by Shaw et al., especially during periods of SMI symptom exacerbations, though some patients disliked the impersonal feel [33]. Notably, there was limited reporting on other potential patient-level barriers such as cognitive impairment, financial constraints, or broader access-related challenges, suggesting these factors may be underexplored in the current primary care intervention literature.

#### b. Staff-level barriers and facilitators.

Hassan et al. identified prior staff experience of working with SMI patients as a facilitator, as it enabled knowledgeable, confident and open interactions with patients, while a lack of SMI knowledge and experience among some staff led to anxiety and negatively impacted intervention delivery [27]. Staff training was identified as a facilitator, with Lawless et al. reporting that nurses generally found the TTIM manual useful for intervention delivery, Hassan et al. that training was essential in increasing staff confidence and competence interacting with SMI patients and Shaw et al. reporting that staff perceived provided training as useful, especially due to addressment of initial education and knowledge disparities [27,28,33]. Inadequate training was seen as a barrier, with Shaw et al. reporting that a lack of training content depth reduced staff confidence in intervention delivery [33]. The use of care plans was viewed as both a barrier and a facilitator by staff, similar to patients. Hassan et al. reported that some staff found written care plan implementation into routine practice difficult and time-consuming, however Lawless et al. mentioned that personalised care plan use helped nurses better understand individual patient’s learning requirements [27,28]. Mental health stigma among staff was another barrier to care delivery, with Hassan et al. reporting that preconceptions among some staff negatively impacted their perceptions of intervention delivery capability, and reduced willingness to participate, though most staff were motivated and had a positive attitude [27].

#### c. Logistical barriers and facilitators.

A key barrier identified across two studies was the need for increased time and resource allocation. Hassan et al. reported that implementation of the PRIMROSE intervention into busy primary care settings resulted in staff concerns and doubts regarding its feasibility, particularly in relation to increased workload, time constraints within routine consultations, and the practicality of integrating longer, intervention-specific appointments into existing schedules [27]. Contextual integration challenges also impaired staff intervention delivery, with staff facing challenges dedicating sufficient time to facilitate patient engagement and accessibility, and fitting longer appointment times around their daily schedules [27]. Similarly, Shaw et al. found that time and resource allocation for PRIMROSE-A was a common staff concern, especially regarding long-term integration into practice outside the trial setting [33]. However, extended appointment times and regular contact were identified as key facilitators, though nurses emphasised that this extra time must be utilised effectively [33]. Further barriers identified by Shaw et al. included problems with reimbursement, general organisation, and communication problems between healthcare and managerial staff, for example with the lack of provision of peer mentor contact details to healthcare staff in certain instances [33]. Lawless et al. identified inconvenient class times, transport limitations, inconsistent attendance, inconsistent peer behavioural modelling due to heterogeneity of symptoms, and challenges contacting patients as logistical barriers to intervention delivery [28]. Facilitative solutions implemented by nurses to tackle such problems were also reported, including changing of class times based on patient feedback, and the nomination of a secondary contact by patients whom nurses can contact if required [28]. Additionally, the availability of external resources, including local referral services, was identified as a facilitator to care by Hassan et al. [27].

### 5. Participant experiences and viewpoints

The experiences and viewpoints of both patients and primary care staff involved in interventions was outlined in four studies, as was feedback for intervention modifications.

#### a. Therapeutic relationship.

The therapeutic relationship between patient and healthcare professionals was reported as an important aspect in four studies, valued by both patients and staff. Lee et al. reported that patients valued psychological space and protected time provided by trained mentors (e.g., healthcare or support professionals), enabling discussion about their concerns, and validating their experiences [29]. PRIMROSE-A also emphasised the fostering of positive therapeutic relationships, addressing additional patient needs and non-judgemental staff attitudes, contributing to positive patient experiences with this intervention [33]. The establishment of positive therapeutic relationships, with long term follow-up care and goal setting was also viewed as positive and rewarding by staff, as reported by Lawless et al. and Shaw et al. [28,33]. Hassan et al. reported that staff understood the value of therapeutic alliances in instilling confidence and trust in patients, which they cultivated, and resulted in increased patient willingness to engage [27].

#### b. Perceived intervention purpose and value.

Clarity of understanding of intervention purpose and value was mentioned in four studies. Hassan et al. reported generally good understanding of the purpose of PRIMROSE among patients, facilitated by clear explanations by general practitioners and information sheet provision [27]. This intervention was also perceived as valuable by staff, as they understood the potential improvements in patients’ physical health and quality of life, and by patients as they recognised the opportunity to improve their own health [27]. Patients also viewed the longer appointments in PRIMROSE as more holistic than usual GP appointments [27]. Shaw et al. mentioned that PRIMROSE-A was greatly valued by patients, especially due to addressment of their social needs during the COVID-19 pandemic through connection with peer educators and carers, though some patients incorrectly understood the intervention to be regarding mental health purely, and not cardiovascular health also [33]. Staff also found the experience of PRIMROSE-A rewarding, for example due to receival of positive patient feedback [33]. Lee et al. reported that patients felt optimistic prior to initiation of WHEEL, potentially fostered by information booklet provision, and appreciated the opportunity to share and learn about weight management in the context of living with SMI [29]. Sajatovic et al. reported that the vast majority of patients surveyed post intervention agreed that TTIM was useful, and that it covered issues most important to them and in general, regarding diabetes self-management in an SMI context [32].

#### c. Feedback for improvement.

Patients feedback for intervention improvement was mentioned in three studies. Lee et al. mentioned that patients recommended peer educator involvement in adjunct support to address feelings of isolation [29]. More structured mentor meetings were also requested to help patients with disorganised thoughts better share their experiences [29]. Requests were also made for further tailoring of intervention information booklets to address individual patient challenges and goals, and for sending of email appointment reminders to patients [29]. Patient preferences for appointment frequency and duration were mixed, with some preferring more frequent and rigid timings, and others more flexible scheduling [29]. Shaw et al. mentioned that some patients desired more follow-up appointments, longer appointments and further peer educator involvement [33]. Hassan et al. mentioned that staff recommended creation of designated timeslots for intervention delivery, to facilitate clinical implementation and longer appointments, with a need for further external resources and staff training also highlighted [27]. Patients also requested better integration of digital technology to track intervention progress [27].

## Discussion

### Key findings

This scoping review identified limited but evolving evidence base for primary care interventions aimed at reducing CVD risk in patients with SMI. Although only ten studies met inclusion criteria, several consistent patterns emerged. Interventions were heterogeneous, but multicomponent and collaborative approaches, particularly those involving nurses, peer workers, or multidisciplinary teams, appeared more promising than single-component interventions. A key finding was the central role of therapeutic relationships and patient engagement, which were consistently associated with improved acceptability and participation. However, significant implementation barriers were evident across studies, particularly time constraints, limited resources, and gaps in staff training, which frequently undermined intervention delivery. Despite some promising intervention components, there was limited evidence of sustained clinical effectiveness, particularly for individual risk factors such as smoking and cholesterol. This highlights an important gap between intervention design and real-world impact in primary care settings.

### Comparison with existing literature

#### Primary care interventions and modified primary care models.

Prior literature reviews and guidelines have identified, evaluated and explored CVD risk management interventions for SMI patients [9,16,34]. In contrast to our study, the aforementioned reviews did not exclusively focus on primary care interventions, by including studies set in secondary care, and they described pharmacological therapies, including metformin for weight management and statins for cholesterol management, which our review did not emphasise. A systematic review was also performed by Osborn et al. to identify pharmacological and behavioural interventions to inform the creation of the PRIMROSE intervention, which two studies in our scoping review covered [27,30,35]. This systematic review mentioned that most included trials were set in secondary care, highlighting a potential lack of research set in primary care, which our review corroborates as only 10 relevant primary care studies published in English between 2015 and 2025 were identified [35].

A study by Murphy et al. theorised a care continuum for CVD risk management in SMI patients, describing strategies such as clinical tool use and modified primary care models, including patient centred medical homes, which our review also identified [26,31,36]. Murphy et al. refers to the value of peer mentors in helping patients engage with chronic condition self-management, which was also identified by four studies in our review [26,28,32,33,36]. Furthermore, the issue of mental health stigma and unconscious bias among staff was identified, as in our review, with exploration of potential solutions, such as setting up of contacts between stigmatised individuals and healthcare workers, though the issue of limited data on methods to address this stigma was highlighted [27,36].

Only one study in our review explored the PCMH primary care model, though existing literature describes another PCMH called SMI PACT with similar features, including tailored SMI care provision, frequent follow-ups, and implementation of a similar collaborative team, excepting peer mentors, but not with cardiovascular risk reduction as a primary outcome [37,38]. SMI PACT was associated with improved overall chronic disease care and positive patient experiences, in part due to considerate and personalised communication, fostering therapeutic relationships [37,38]. This promise of the PCMH model in chronic disease management echoes Gertner et al. in our review, and the value of therapeutic relationships was a consistent finding across four studies in our review [26–29,33].

#### Qualitative interventional aspects.

Qualitative studies by Jakobs et al. and Burton et al. identified barriers and facilitators to existing CVD risk management for SMI patients in primary care, with similar features emerging in the context of novel interventions in our review [39,40]. For example, facilitators such as staff training and doctor-patient relationships, and barriers such as lack of staff knowledge, increased workload and mental health stigma among staff, also emerged in our review [27,29,32,33,39,40]. While prior literature has highlighted lack of information exchange between primary care and psychiatric services [39,40], this review more frequently identified communication challenges within primary care teams and between clinical and managerial staff [26,28,32,33]. This distinction suggests that both system-level integration issues and organisational or interpersonal communication barriers may impact implementation.

Burton et al. also described patient and staff identified strategies for care improvement, including enhanced care continuity and achievable goal setting [40]. Similar strategies also emerged in our review, both as considerations implemented in included studies, such as lengthened appointment times and personalised goal setting in PRIMROSE, and as participant feedback for intervention improvement, such as a desire for longer term follow-ups identified by Shaw et al. [27,30,33]. Costa et al. also found that primary care providers desired more information on suitable local services, such as weight loss referral programmes, echoing findings from Hassan et al. in our review [27,41].

### Strengths and limitations

Strengths of this scoping review include adherence to the Arksey and O’Malley framework and Prisma-ScR checklist, which provided a standardised approach from identification of the research question to literature selection and analysis [20,23]. However, this study also has several limitations. It is possible that not all relevant articles were identified by our search strategy, included databases, or exploratory searching. Relevant studies not published in the English language were also not identified. Quality assessment of included studies was not performed, as is convention with scoping reviews, which should be considered when adjudging the results and conclusions of this study. The applicability of our findings may also be limited to primary healthcare structures similar to the countries of our included studies. Pharmacological interventions, interventions for paediatric populations, or CVD screening interventions were also not explored by design in this study, potentially limiting the breadth of this review. However, the scoping review structure was suitable for this context due to the exploratory nature of the research question, as it facilitated the structured identification and description of relevant literature [20].

### Implications for research, policy and practice

This review highlights a clear gap in the evidence base for primary care interventions targeting CVD risk in people with SMI but also identifies several important directions for future work. A key implication is the value of multidisciplinary, team-based care models in primary care. Interventions incorporating nurses, peer workers, and other non-physician roles appeared to enhance patient engagement and support behavioural change, suggesting that addressing CVD risk in SMI populations may require redistribution of care beyond traditional GP-led models.

However, the findings also highlight that implementation challenges remain a major barrier. Time constraints, limited resources, and insufficient staff training were consistently reported, indicating that intervention success depends not only on design but also on the capacity of primary care systems to deliver them. Importantly, despite longstanding policy initiatives such as incentivised physical health monitoring for people with SMI in primary care, there is limited evidence of meaningful improvement in outcomes. This suggests that screening alone is insufficient, and that without adequate follow-up care, training, and system-level support, such initiatives may have limited impact.

Another important gap identified in this review is the lack of consideration of population diversity. None of the included studies explicitly examined how factors such as cultural and linguistic diversity (CALD), gender, or socioeconomic status may influence engagement with or effectiveness of interventions. Given known disparities in both mental health outcomes and cardiovascular risk, future research should prioritise inclusive study designs and tailored interventions that account for the needs of diverse populations.

Further research on patient needs, considerations and experiences, and clinical and cost effectiveness of interventions could also be of value, as only four studies emphasised participant experiences, and four tested clinical effectiveness, of which only one also emphasised cost efficiency [25,27–33]. Interventions for paediatric populations may also need to be explored, as studies on those populations were outside the scope of our review.

## Conclusion

This scoping review demonstrates that, despite growing recognition of the cardiovascular health needs of people with severe mental illness, the evidence base for effective primary care interventions remains limited and fragmented. While promising approaches particularly those involving multidisciplinary collaboration, patient engagement, and tailored support have been identified, their impact is constrained by persistent implementation challenges. Improving cardiovascular outcomes in this population will likely require not only the development of new interventions, but also system-level changes to primary care delivery, including increased resources, enhanced staff training, and better integration across services. Without addressing these structural barriers, primary care is unlikely to achieve meaningful reductions in cardiovascular risk for people with SMI.

## Supporting information

S1 File PRISMA-ScR checklist. (PDF)

## Abbreviation:

SMI: severe mental illness
CVD: cardiovascular disease
PRISMA-ScR: Preferred Reporting Items for Systematic Reviews and Meta-analyses Extension for Scoping Reviews
UK: United Kingdom
GP: general practitioner
NICE: National Institute for Health and Care Excellence
QOF: quality and outcomes framework
HSE: health service executive
PE: provider education
CHW: community health worker
EPC: enhanced primary care
TTIM: Targeted Training and Illness Management
WHEEL: weight change for people with serious mental illness
TAU: treatment-as-usual
CDS: clinical decision support
DM: diabetes mellitus
RCT: randomised controlled trial
PCMH: patient-centred medical home
HbA1c: glycated haemoglobin
LDL: low density lipoprotein
PCP: primary care provider
CALD: cultural and linguistic diversity
